# Multiband Quasi-Yagi Antenna with Frequency-Selective Multi-Branch Directors for Sub-6 GHz Applications

**DOI:** 10.3390/s26123631

**Published:** 2026-06-07

**Authors:** Dokhyl AlQahtani, Faroq Razzaz, Saud M. Saeed

**Affiliations:** 1 Electrical Engineering Department, College of Engineering, Prince Sattam Bin Abdulaziz University, Al-Kharj 16278, Saudi Arabia; dm.alqahtani@psau.edu.sa; 2 Prince Sultan Defense Studies and Research Center (PSDSARC), Riyadh 13322, Saudi Arabia; saud.binsaeed@psdsarc.org.sa

**Keywords:** high-gain antenna, multiband antenna, multibranch line director, quasi-Yagi, sub-6 GHz

## Abstract

This paper presents a novel design of a high-gain, low-profile multiband quasi-Yagi antenna. The proposed antenna will operate in the 2.45 GHz, 3.60 GHz, and 5.80 GHz frequency bands. The proposed antenna consists of a primary driven dipole printed on the sides of a substrate, two parasitic elements, and a new branch line director. The main dipole element is utilized to generate the first frequency band. The two parasitic elements added near the driven dipole excite the last two frequency bands. The proposed antenna is appropriate for multiband applications due to its directional radiation patterns and front-to-back ratios, which exceed 13.4 dB for all frequency operating bands. The single-branch line director antenna realizes gains of 6.7, 7.5, and 7.4 dBi at 2.45, 3.6, and 5.8 GHz, respectively. When the number of branch line directors increases, the antenna’s gain increases over all the operating frequency bands. The realized gains with five branch line directors are 10.1, 11.8, and 11.9 dBi at 2.45, 3.6, and 5.8 GHz, respectively. Moreover, a 2 × 1 MIMO configuration is also demonstrated, achieving inter-element isolation greater than 20 dB at 2.45 GHz and 30 dB at 3.60 and 5.80 GHz, confirming the antenna’s suitability for 5G, Wi-Fi, and IoT sub-6 GHz applications.

## 1. Introduction

Sub-6 GHz wireless systems, such as 5G, Wi-Fi, and IoT applications, require antennas capable of operating at multiple frequency bands within a single platform. In practical implementations, using separate antennas for each band is often not feasible due to space limitations and increased system complexity. Therefore, multiband antennas have become an essential solution for supporting multi-service communication while maintaining a simple structure. Microstrip antennas are widely used in such systems because of their low profile, ease of fabrication, and compatibility with planar circuits [1,2,3].

Among planar antenna structures, the printed quasi-Yagi antenna has been widely adopted due to its directional radiation characteristics and relatively simple design. Various approaches have been proposed to extend quasi-Yagi antennas to multiband operation. For example, dual-band designs have been achieved by modifying the driver and director structures [4,5,6,7,8], while tri-band operation has been realized using parasitic elements and modified feeding techniques [9,10,11,12,13,14]. Additional multiband implementations based on parasitic coupling and metamaterial-inspired structures have also been reported [15,16,17]. In addition, filtering quasi-Yagi antennas have been introduced to improve frequency selectivity and radiation performance [18,19,20].

Although several multiband quasi-Yagi antennas with branched or multiresonant director structures have been reported, most existing designs employ coupled director branches that simultaneously affect multiple operating bands. As a result, the electromagnetic behavior of the director changes significantly with frequency. In some cases, a director optimized for a lower frequency band may become electrically long at higher frequencies and behave similarly to a reflector element, which can degrade antenna directivity and front-to-back ratio. In addition, strong interaction between director branches may introduce undesired coupling effects that reduce gain stability across the operating bands.

In this work, a frequency-selective multi-branch line director (MBLD) is proposed to reduce these limitations. Unlike conventional branched directors, each branch in the proposed structure is dimensioned to mainly control a specific operating frequency band. This approach improves current distribution control and reduces unwanted interactions between the operating bands, leading to more stable gain performance and improved radiation characteristics.

Despite these developments, achieving stable and high gain across multiple frequency bands remains a key challenge. In conventional multiband quasi-Yagi designs, the director element does not behave consistently across frequencies. A director optimized for a lower band can become electrically long at higher frequencies and act as a reflector, which degrades the radiation pattern and reduces the front-to-back ratio [21]. This issue makes it difficult to maintain uniform performance across all operating bands.

Another limitation is that gain improvement is often more noticeable at higher frequencies, while the performance at lower frequencies remains constrained. This is mainly due to the larger physical size required for effective radiation at lower bands [22]. During the design process, it was also observed that increasing the number of directors does not necessarily improve performance across all frequencies. In some cases, additional elements introduce stronger coupling, which complicates impedance matching and reduces overall efficiency, particularly in the mid-band region.

Increasing the electrical length of directors can improve radiation performance at lower frequencies, but this often leads to undesired interactions at higher bands. As a result, designers are required to carefully balance structural dimensions and electromagnetic behavior to avoid performance degradation across the operating spectrum.

To address these challenges, a multi-branch line director (MBLD) is proposed in this work. Unlike conventional single-element directors, the proposed structure uses multiple branches, each designed to operate at a specific frequency band. This approach allows better control of current distribution and reduces unwanted interactions between bands, leading to improved gain performance.

Based on this concept, a triband quasi-Yagi antenna operating at 2.45, 3.60, and 5.80 GHz is designed and fabricated. The antenna consists of a printed dipole driver, a reflector, two parasitic elements, and the proposed MBLD structure. The main dipole is used to generate the lowest frequency band, while the parasitic elements are responsible for exciting the higher-frequency bands. In addition, configurations with multiple branch line directors are investigated to evaluate their effect on gain performance. A 2 × 1 MIMO configuration is also implemented to assess inter-element coupling and system performance. The results demonstrate that the proposed design achieves improved gain behavior across all operating bands while maintaining directional radiation characteristics.

## 2. Antenna Design and Structure

### 2.1. Proposed Antenna Structure

**Figure 1:**
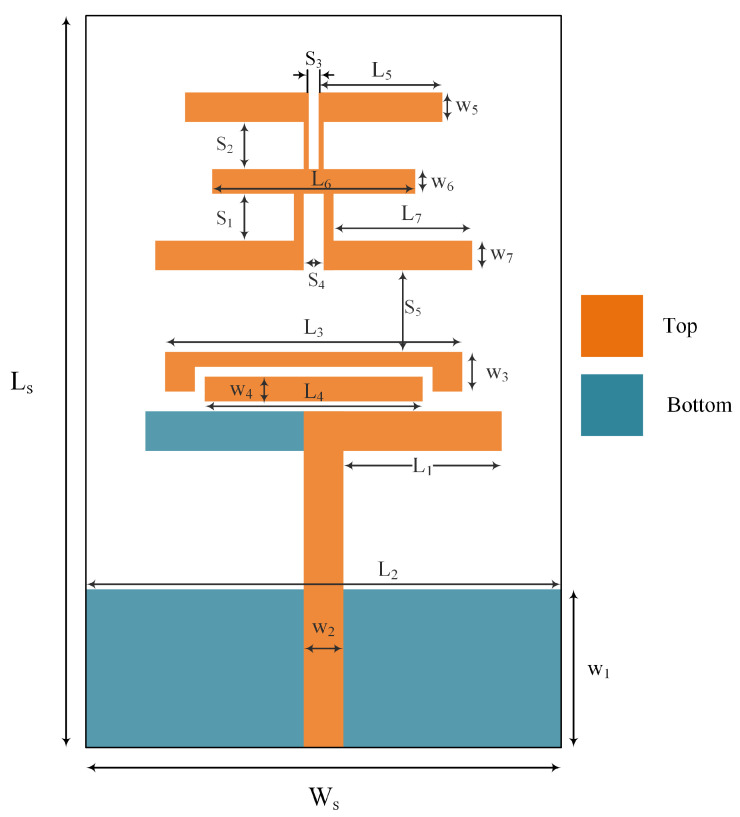
Structure of the proposed multiband printed Yagi–Uda antenna.

The structure of the proposed multiband printed Yagi–Uda antenna is illustrated in Figure 1. This antenna comprises the main printed dipole, a reflector, two parasitic elements, and one multibranch line director. The primary printed dipole is printed on the substrate on both sides and is intended to function at the first frequency band. By introducing parasitic elements close to the main dipole, the second and third frequency bands are produced. The frequency bands are determined by selecting the length, width, and gap between the main printed dipole and the parasitic element. As indicated in Figure 1, the more prominent parasitic element with L_3_ and W_3_ generates the second frequency band, that at 3.6 GHz. The gap between this parasitic element and the main printed dipole is 2.02 mm. The minor parasitic element with L_4_ and W_4_ generates the third frequency band at 5.8 GHz. The gap between this parasitic element and the main printed dipole is 1.37 mm. Furthermore, the gap between these two parasitic elements is 0.45 mm. Table 1 lists optimized values of the proposed triband quasi-Yagi antenna’s remaining parameters.

### 2.2. Design Methodology

**Figure 2:**
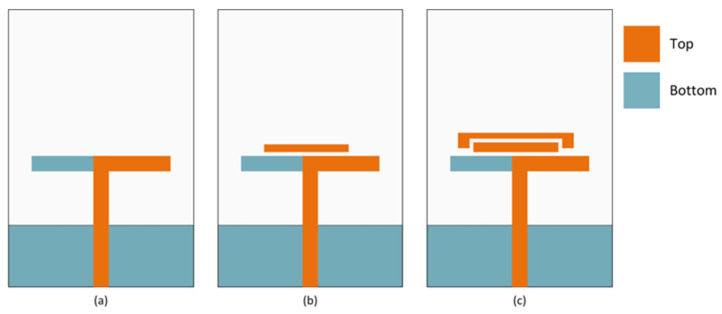
Proposed multiband printed Yagi–Uda antenna design steps. (**a**) Printed dipole with reflector. (**b**) Printed dipole with reflector and one parasitic element. (**c**) Printed dipole with reflector and two parasitic elements.

**Figure 3:**
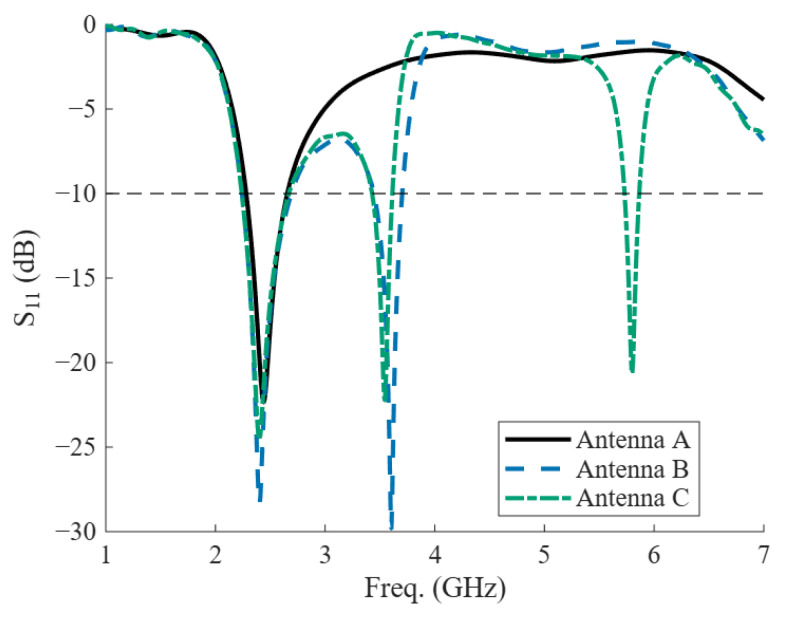
Simulated scattering parameter, S_11_, for the antennas in Figure 2. (The gray dashed line indicates the −10 dB reference level).

The steps involved in designing the proposed multiband antenna are depicted in Figure 2. First, a traditional printed dipole is made to function at 2.45 GHz, which is the lowest frequency range. As seen in Figure 2a, this dipole antenna comprises two metallic arms printed on both sides of a dielectric substrate. Additionally, to enhance the reflectivity of this antenna, a metallic ground is carefully designed and introduced at the bottom of the dielectric substrate. Secondly, to excite the second frequency band of 3.6 GHz, a parasitic metallic element of suitable size is added close to the main dipole on the top of the dielectric substrate, as depicted in Figure 2b. For improved performance in both frequency bands, the spacing between the additional parasitic element and the main dipole element is optimized. In the third design step, another metallic parasitic element of suitable length and width is added to excite the third frequency band at 5.8 GHz. However, this parasitic element interferes with the parasitic element introduced in the second step. Hence, an optimized gap between these parasitic elements is then introduced to ensure the excitation of the required frequency bands, as shown in Figure 2c. The scattering parameter of these antennas, S11, is depicted in Figure 3.

**Figure 4:**
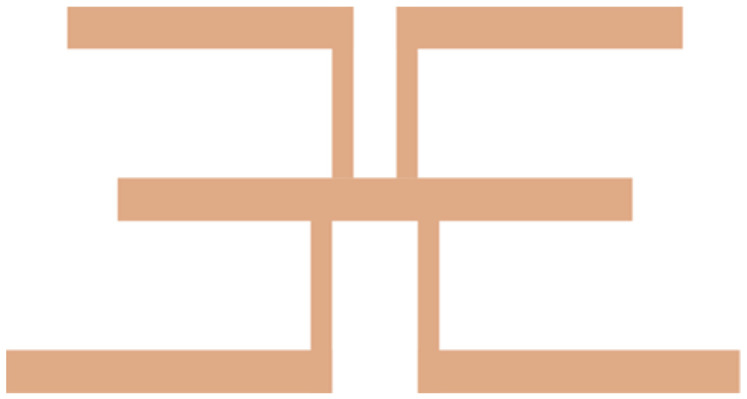
Proposed multibranch line director.

To improve antenna directivity and gain, the directors in traditional Yagi–Uda antennas are frequently made for a specific frequency. Directors meant for lower frequencies, however, will function as reflectors for higher frequencies if the antenna is made for multiband operation, which will lower the directivity and gain of the device. In this work, the multibranch line director depicted in Figure 4 was designed to overcome this problem and operate at 2.45, 3.6, and 5.8 GHz. To improve antenna directivity and gain over the three operating bands, a frequency-selective multibranch line director (MBLD) is introduced, as shown in Figure 4. Unlike conventional multiresonant directors, where all branches strongly interact over the entire frequency range, the proposed structure is designed such that each branch mainly contributes to a specific operating band. The effective electrical length of each branch is approximately related to the guided wavelength corresponding to its target frequency band. The lower branch mainly affects the first operating band at 2.45 GHz, while the upper and middle branches primarily contribute to the second and third operating bands at 3.6 GHz and 5.8 GHz, respectively. By carefully optimizing the branch lengths and spacing between the branches, the proposed MBLD reduces undesired inter-band coupling and improves gain stability across the operating frequencies.

**Figure 5:**
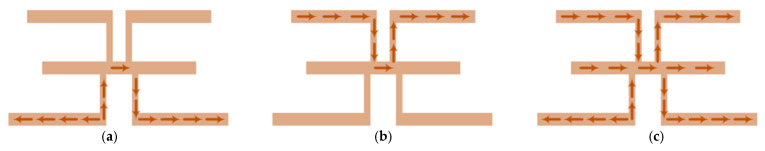
Surface current distributions demonstrating the frequency-selective behavior of the proposed multibranch line director (MBLD): (**a**) 2.45 GHz, (**b**) 3.6 GHz, and (**c**) 5.8 GHz. (The arrows indicate the direction of the surface current flow).

The surface current distributions shown in Figure 5 confirm this behavior. At 2.45 GHz, the current is mainly concentrated along the lower branch line director. At 3.6 GHz, stronger current concentration appears on the upper branch line. At 5.8 GHz, the current becomes dominant on the middle branch line, while weaker current distributions are observed on the remaining branches.

## 3. Results and Discussions

In this section, we describe the design, simulation, fabrication, and testing of the proposed antenna. Full-wave electromagnetic simulations were carried out using CST (Dassault Systèmes, Vélizy-Villacoublay, France) Microwave Studio. To this end, we utilized a Roger RO-3003 (Rogers Corporation, Chandler, AZ, USA) dielectric substrate with a thickness of 1.52 mm, a dielectric constant of 3, and a loss tangent of 0.001. A laser etching system was used to fabricate the proposed multiband antenna.

The reflection coefficient measurements were carried out using a calibrated Rohde & Schwarz ZNB20 Vector Network Analyzer (VNA) (Rohde & Schwarz GmbH & Co. KG, Munich, Germany) operating from 100 kHz to 20 GHz. Standard SOLT calibration was performed prior to the measurements to minimize cable and connector effects.

**Figure 6:**
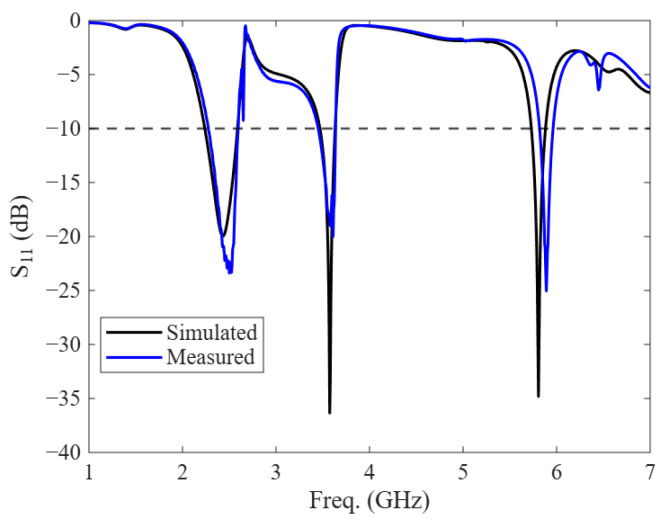
Simulation and measurement S_11_ of the proposed antenna with a single multibranch line director. (The gray dashed line indicates the −10 dB reference level).

**Figure 7:**
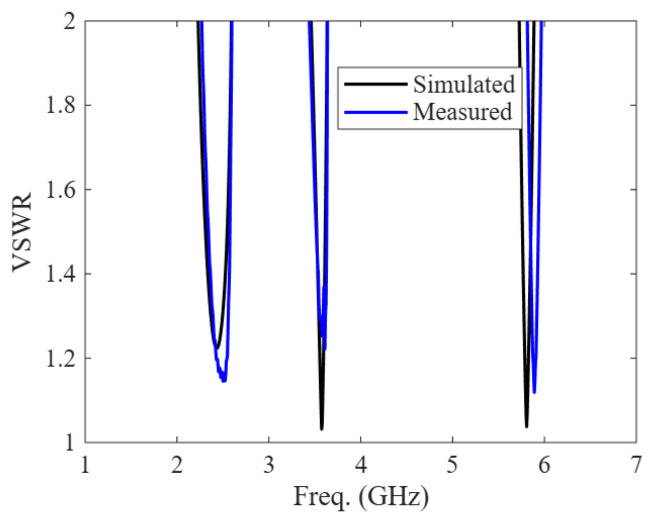
Simulation and measurement VSWR of the proposed antenna.

Figure 6 shows the simulation and measurement outcomes for the reflection coefficient (S11) of the proposed antenna with a single-branch line director. The simulation and measurement outcomes of the different frequency bands show good agreement. The measurement outcomes indicate that the bandwidth of the −10 dB reflection coefficient for the first frequency band is 312 MHz (2.278–2.590 GHz); for the second frequency band it is 180 MHz (3.454–3.634 GHz), and for the third frequency band it is 138 MHz (5.818–5.956 GHz). Furthermore, as shown in Figure 7, the developed antenna has a simulation and measurement VSWR that is significantly better than 1.5.

**Figure 8:**
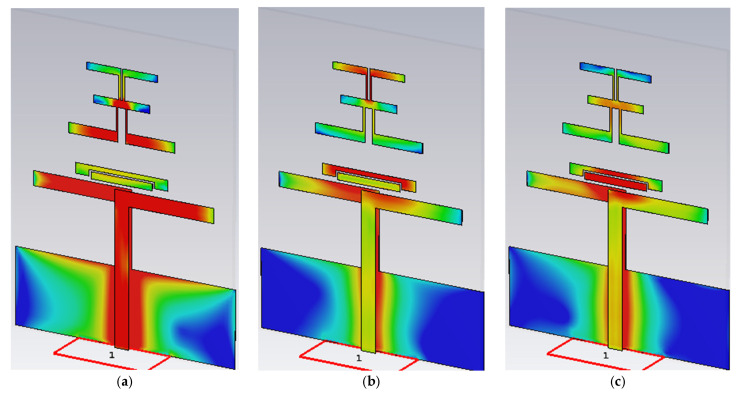
Surface current distributions of the proposed multiband quasi-Yagi antenna at: (**a**) 2.45 GHz, (**b**) 3.6 GHz, and (**c**) 5.8 GHz, illustrating the dominant current paths at different operating bands.

The surface current distribution simulation results at the operating frequency bands are shown in Figure 8. The main printed dipole driver and the low branch line director have the highest current density within the first frequency band, 2.45 GHz, as shown in Figure 8a. The reflector also requires relatively higher current densities to achieve the intended antenna gain. The distribution of current is concentrated in the long parasitic element and the upper branch line director in the second frequency band, as depicted in Figure 8b. Furthermore, at 5.8 GHz, the flow of current is the most pronounced in the short parasitic element and the middle branch line director and less so in the remaining branch lines, as depicted in Figure 8c.

Table 2 summarizes the simulation outcomes of the designed antenna’s gain and radiation effectiveness over the three operating frequency bands. Adding a single multibranch line director increases the gain by almost 1 dBi, 2 dBi, and 2 dBi at 2.45, 3.6, and 5.8 GHz, respectively. The gain enhancement introduced by the proposed MBLD becomes more noticeable at higher operating frequencies due to the improved end-fire current distribution provided by the frequency-selective director branches.

To further illustrate this, a conventional single-frequency director tuned to 5.8 GHz was simulated for comparison. At this frequency, the director provides a gain improvement comparable to the MBLD. However, this element cannot be replicated at 2.45 GHz or 3.6 GHz, since a director designed for these lower frequencies would be physically longer and therefore electrically long at 5.8 GHz, causing it to act as a reflector and degrade both the radiation pattern and the front-to-back ratio. This confirms that a single conventional director cannot serve all three bands simultaneously, which is the fundamental limitation that the proposed MBLD is designed to overcome.

**Figure 9:**
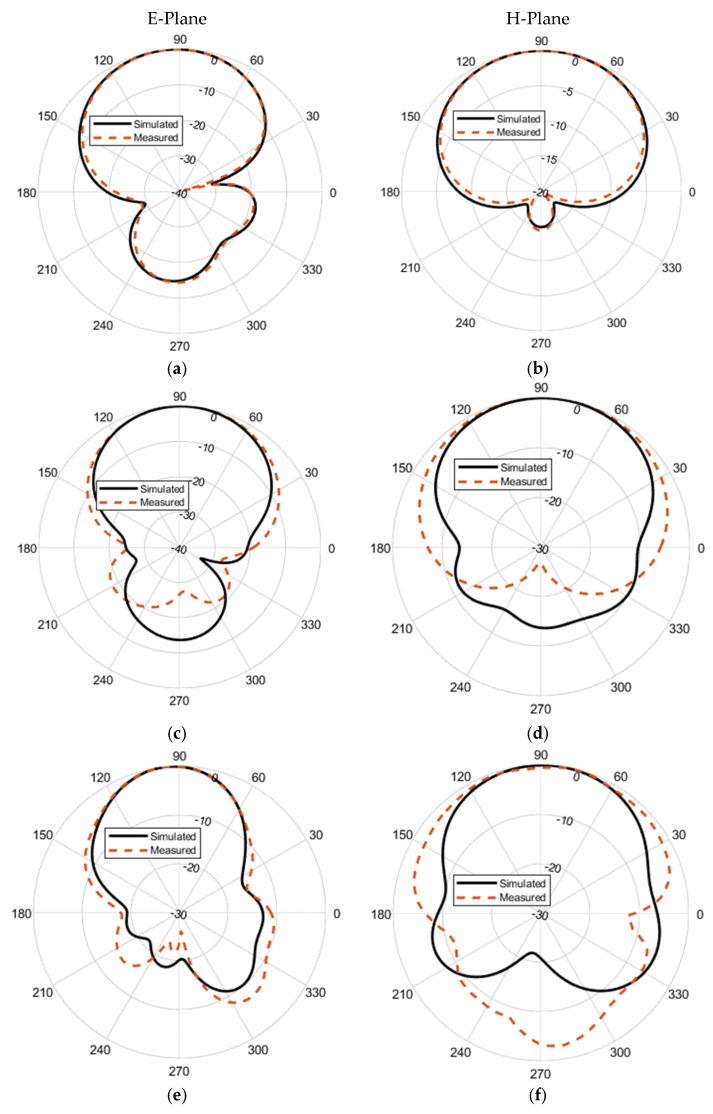
The normalized E-plane and H-plane radiation patterns of the proposed antenna: (**a**,**b**) 2.45 GHz, (**c**,**d**) 3.6 GHz, and (**e**,**f**) 5.8 GHz.

Figure 9 shows the E-plane and H-plane’s normalized simulation and measurement radiation patterns across the three operational frequency bands. The main beams point toward the end-fire, and the front-to-back ratios are greater than 13 dB across all specific operating frequency bands. In both planes, there is good agreement between the simulated radiation patterns and experimental measurements.

### 3.1. Parametric Study

**Figure 10:**
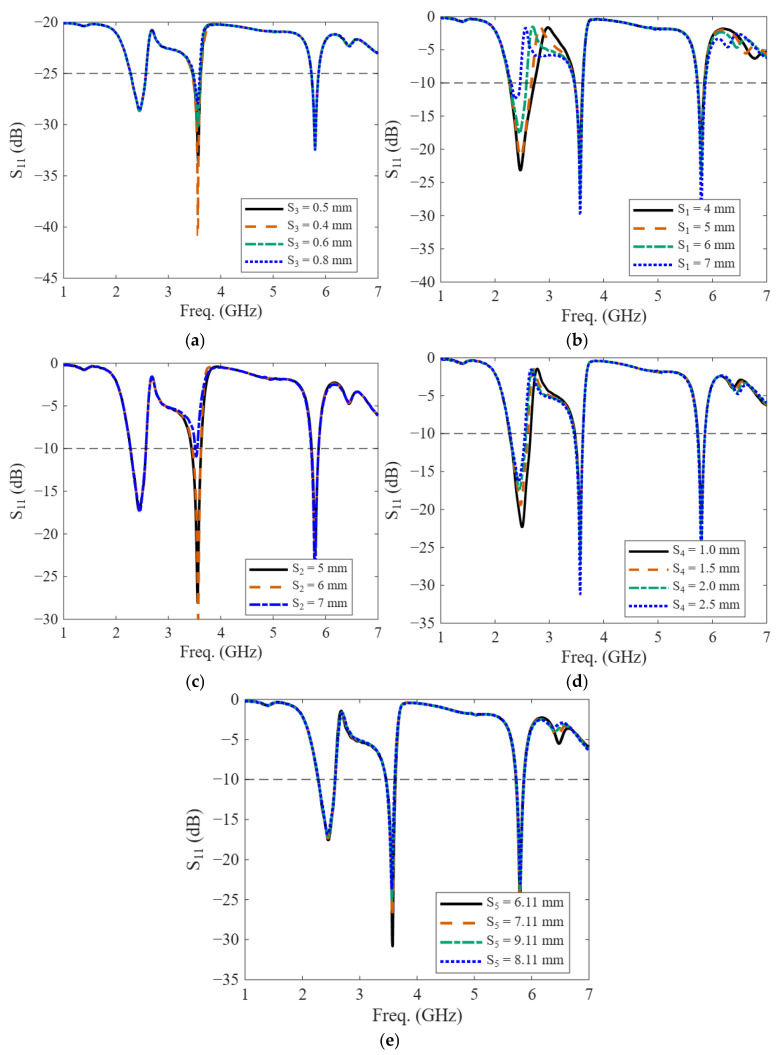
Parametric study of the design parameters of the proposed antenna. (**a**) S_3_. (**b**) S_1_. (**c**) S_2_. (**d**) S_4_. (**e**) S_5_.

In this section, we examine the effect of various design factors on the performance of the developed antenna. Figure 10a illustrates the impact of S_3_ on the performance of the proposed antenna. It is clear that the increase in S_3_ affects the matching impedance at the second frequency band at 3.6 GHz. Figure 10b depicts the effect of S_1_ on the proposed antenna’s performance. Notably, increasing S_1_ significantly affects the matching impedance at the first frequency band, (2.45 GHz). Figure 10c illustrates the effect of the parameter S_2_ on the scattering parameters of the developed antenna. This parameter directly affects the matching impedance at the second frequency band (3.6 GHz). In addition, it emerges that changing the design parameter S_4_ has an effect on the matching impedance at the first frequency band, as demonstrated in Figure 10d. Furthermore, changing the design parameter S_5_ has a pronounced effect on the proposed antenna scattering parameter, as illustrated in Figure 10e.

### 3.2. Increasing Number of Multibranch Line Directors

**Figure 11:**
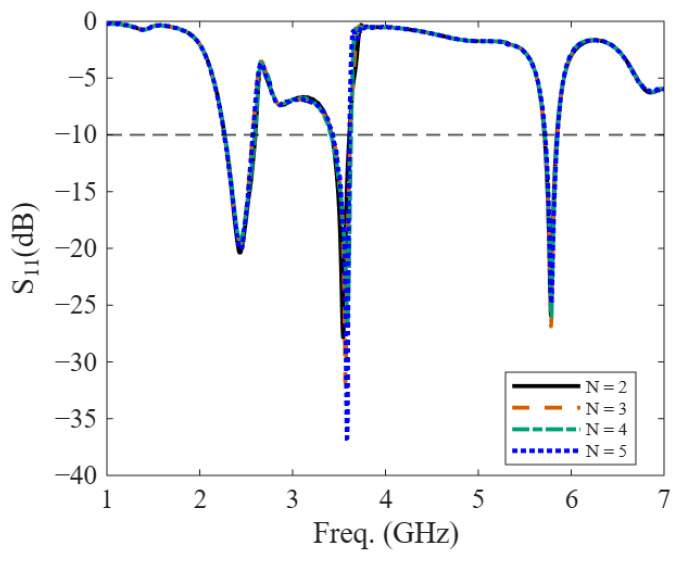
The scattering parameter of the proposed antenna versus the number of multibranch line directors. (The gray dashed line indicates the −10 dB reference level).

**Figure 12:**
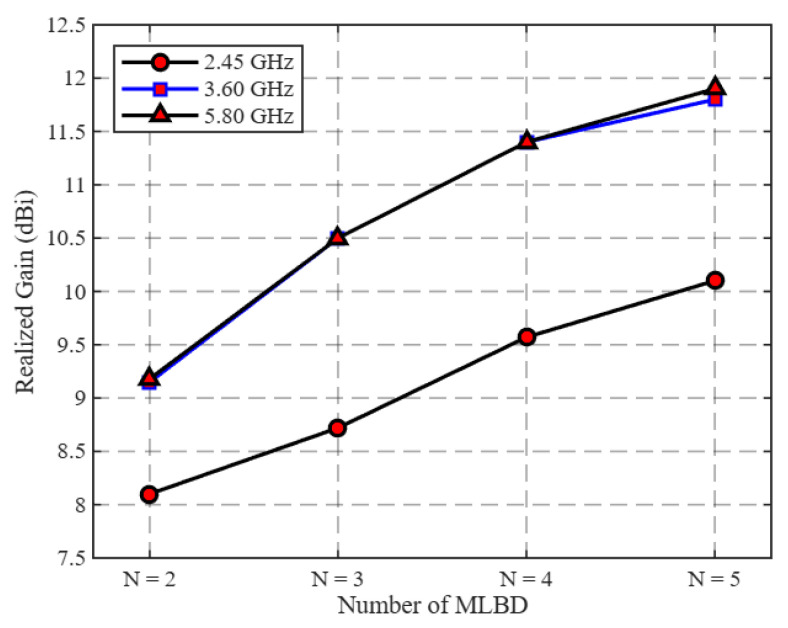
The gain realized by the proposed antenna with different numbers of multibranch line directors.

This section investigates multiband printed Yagi–Uda antennas with different numbers of multibranch line directors. Figure 11 demonstrates the scattering parameter S_11_ of the proposed multiband antenna with various numbers of multibranch line directors. It is clear that the number of multibranch line directors does not affect the matching impedance of the proposed antenna. Table 3 lists the degrees of gain resulting from simulations using the proposed multiband antenna with a different number of multibranch line directors. The realized gain significantly increases as the number of directors increases, as shown in Figure 12. (The gray dashed line indicates the −10 dB reference level).

**Figure 13:**
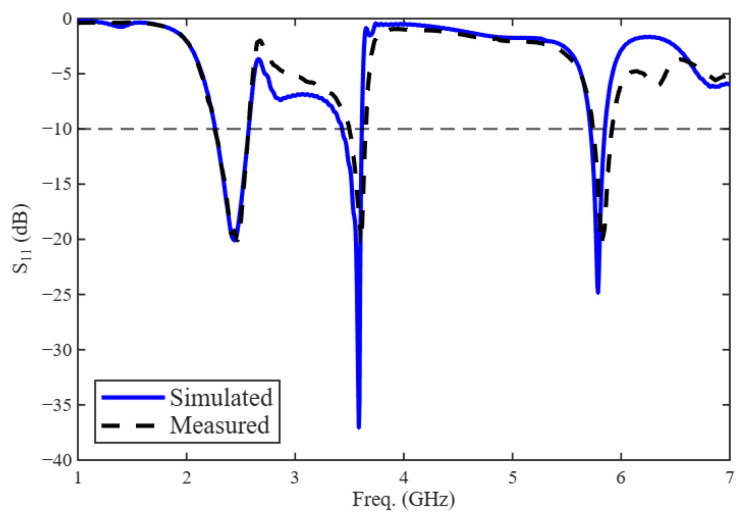
Simulated and measured S_11_ of the proposed antenna with five branch line directors. (The gray dashed line indicates the −10 dB reference level).

**Figure 14:**
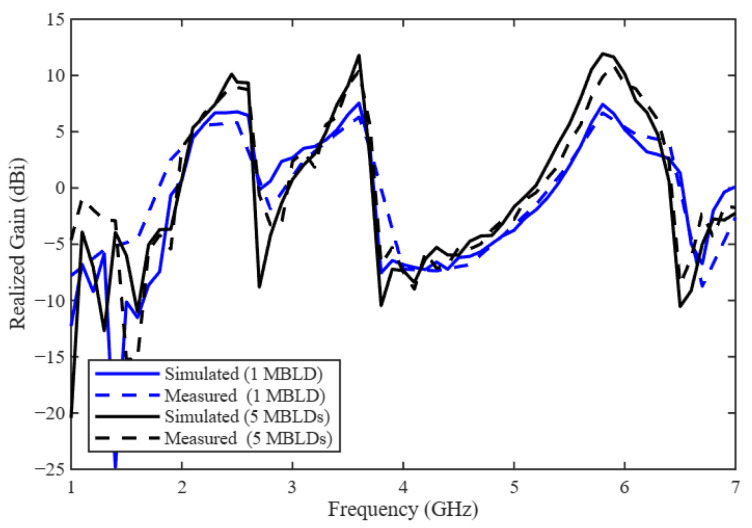
Simulated and measured realized gain of the proposed multiband quasi-Yagi antenna versus frequency.

Figure 13 illustrates both the simulated and measured results of the reflection coefficient S11 of a multiband antenna with five multibranch line directors. The measurement results indicate that the bandwidth of the −10 dB reflection coefficient for the first frequency band is 300 MHz (2.26–2.56 GHz); for the second frequency band, it is 120 MHz (3.52–3.64 GHz), and for the third frequency band, it is 150 MHz (5.76–5.91 GHz). Figure 14 presents the simulated and measured realized gain of the proposed multiband quasi-Yagi antenna. Good agreement between the simulated and measured results is observed across the three operating bands. Minor discrepancies are mainly attributed to fabrication tolerances, connector losses, and measurement uncertainties at higher frequencies. The measured realized gain values are slightly lower than the simulated values due to conductor loss, dielectric loss, and connector effects.

### 3.3. The 2×1 MIMO Antenna

**Figure 15:**
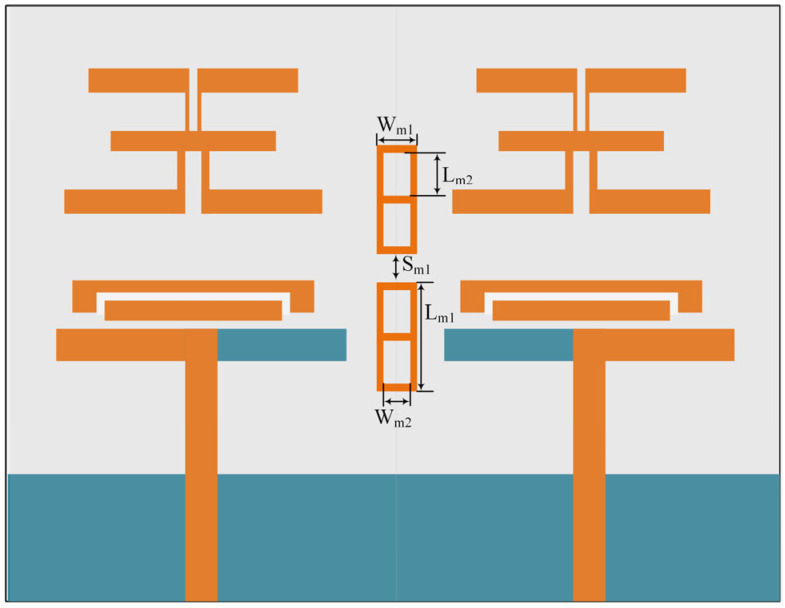
The proposed 2×1 MIMO antenna.

In this section, we investigate the usage of the proposed multiband printed Yagi–Uda antennas as MIMO antennas to increase the data transfer rate and improve the overall system performance, as shown in Figure 15. The structures between the two antennas are utilized to decrease mutual coupling among the proposed 2×1 MIMO antennas. The proposed decoupling structure reduces mutual coupling by suppressing the propagation of surface currents between adjacent antenna elements. The inserted structure modifies the current distribution in the coupling region, which helps reduce undesired coupling and improve the isolation between the antenna elements across the operating bands.

**Figure 16:**
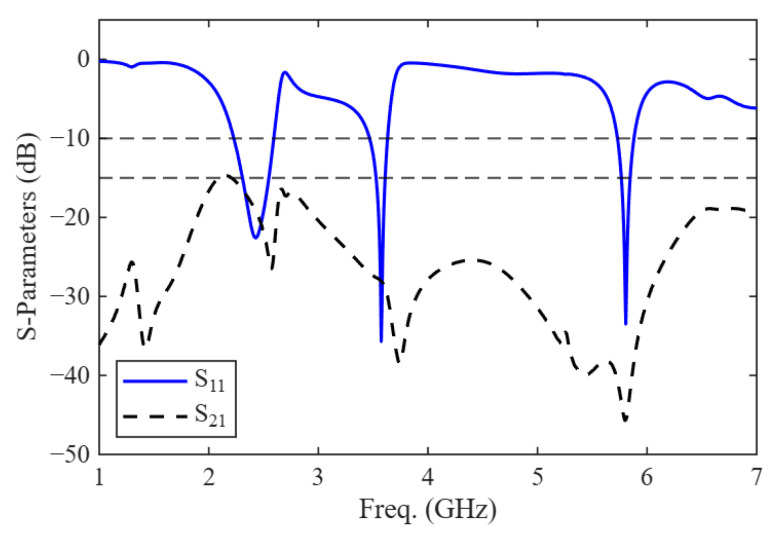
Simulated scattering parameters of the proposed 2×1 MIMO antenna without mutual coupling reduction.

**Figure 17:**
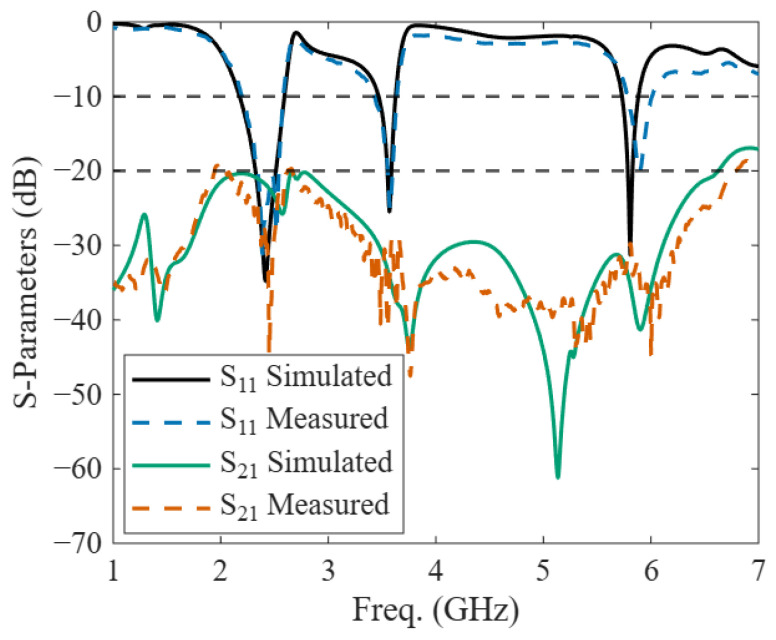
Simulated and measured scattering parameters of the proposed 2×1 MIMO antenna.

Table 4 lists the optimized parameters of the proposed mutual coupling reduction structure. Figure 16 presents the simulated scattering parameters of the proposed 2×1  MIMO antenna without the mutual coupling reduction structure. Less than 15 dB isolation is present in the first operating band, while less than 25 dB isolation is achieved in the other two operating bands. After adding the proposed decoupling structure, the isolation between the antenna elements is significantly improved, as demonstrated in Figure 17. The simulated and measured results of the developed two-element MIMO antenna are presented in Figure 17. In the first operating band at 2.45 GHz, the mutual coupling remains below 20 dB, while in the remaining two operating bands it is approximately below 30 dB.

To further evaluate the MIMO performance, the Envelope Correlation Coefficient (ECC) is calculated using the S-parameter method as follows [23]:

(1) $$\rho_{e}=\frac{{\left|S_{11}^{*}S_{12}+S_{21}^{*}S_{22}\right|}^{2}}{\left(1-{\left|S_{11}\right|}^{2}-{\left|S_{21}\right|}^{2}\right)\left(1-{\left|S_{22}\right|}^{2}-{\left|S_{12}\right|}^{2}\right)}$$

The Diversity Gain (DG) is then obtained as [23]:

(2) $$DG=10\sqrt{1-{\left|\rho_{e}\right|}^{2}}$$

Table 5 summarizes the simulated and measured ECC and DG values of the proposed 2×1 MIMO antenna across the three operating bands. The computed ECC values remain below 0.001 across all three operating bands, which is significantly lower than the acceptable threshold of 0.05. The corresponding DG values remain close to 10 dB, confirming the suitability of the proposed 2×1 MIMO configuration for high-diversity wireless communication systems.

**Figure 18:**
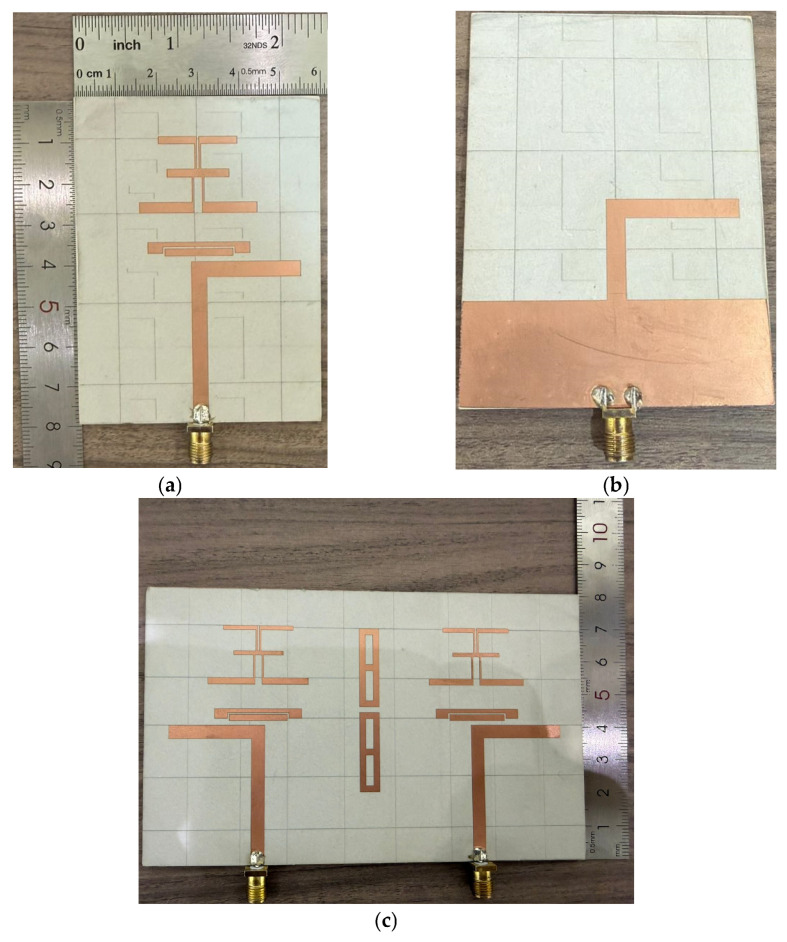
Photograph of the fabricated antennas: (**a**) single MBLD (top); (**b**) single MBLD (bottom); (**c**) 2×1 MIMO antenna.

Photographs of the fabricated antennas with a single multibranch line director, five multibranch line directors, and a 2×1 MIMO antenna are presented in Figure 18.

The multiband antenna proposed in this work can be compared with other multiband antennas found in the literature. Table 6 shows that the proposed antenna provides the highest gain of the techniques detailed in the literature. Moreover, it attains a high front-to-back ratio and can be designed and fabricated easily.

**Figure 19:**
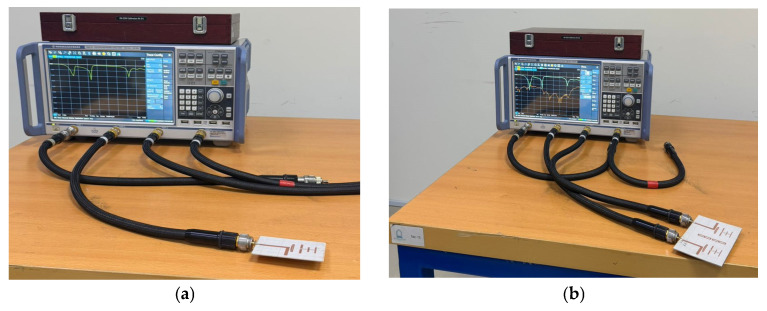
Measurement setup of the fabricated antennas: (**a**) single MBLD antenna and (**b**) 2 × 1 MIMO antenna.

The experimental measurement setups for the fabricated single MBLD antenna and the 2 × 1 MIMO antenna are presented in Figure 19.

## 4. Conclusions

A novel multiband quasi-Yagi antenna with multi-branch line directors has been presented. The frequency-selective MBLD structure enables independent gain optimization at 2.45, 3.60, and 5.80 GHz, overcoming the limitations of conventional multiband Yagi antennas. With five directors, measured realized gains of 10.1, 11.8, and 11.9 dBi are achieved with FBRs ≥ 13 dB and impedance bandwidths of 300, 120, and 150 MHz, respectively. The 2×1 MIMO configuration with |S21| ≤ −20 dB at 2.45 GHz and ≤−30 dB at 3.60/5.80 GHz further confirms the suitability of the proposed antenna for 5G, Wi-Fi, and IoT applications. The proposed frequency-selective MBLD concept can also be applied to other multiband directional antenna structures requiring compact size and stable gain performance across multiple operating bands. Owing to its compact size and directional radiation characteristics, the proposed antenna is suitable for integration into multiband wireless terminals, IoT gateway platforms, and compact wireless sensor nodes operating in Sub-6 GHz environments.

## Figures and Tables

**Table 1:** Proposed antenna dimensions.

Parameter	Value (mm)	Parameter	Value (mm)	Parameter	Value (mm)
L_1_	23.10	w_1_	20.0	S_1_	6.06
L_2_	60.00	w_2_	3.81	S_2_	6.06
L_3_	25.08	w_3_	2.75	S_3_	0.50
L_4_	16.75	w_4_	1.80	S_4_	1.50
L_5_	9.37	w_5_	1.80	S_5_	7.11
L_6_	15.19	w_6_	2.00	L_s_	80.0
L_7_	13.41	w_7_	2.63	W_s_	60.0

**Table 2:** Realized gain and radiation efficiency of the proposed antenna with and without a multibranch line director.

Frequency (GHz)	Gain (dBi)			Radiation Efficiency with MBLD (%)	
	Without Director	Conventional Single Director (5.8 GHz)	with MBLD	Without Director	with MBLD
2.45	5.92	5.9	6.73	97.8	98.2
3.60	5.75	5.75	7.52	96.9	96.2
5.80	5.73	7.12	7.43	93.9	95.3

**Table 3:** Gain and radiation efficiency realized with different numbers of multibranch line directors.

Frequency (GHz)	Gain (dBi)				Radiation Efficiency (%)			
	N = 2	N = 3	N = 4	N = 5	N = 2	N = 3	N = 4	N = 5
2.45	8.10	8.72	9.57	10.1	99.51	98.83	98.07	98.8
3.60	9.15	10.5	11.4	11.8	97.08	96.15	96.24	97.7
5.80	9.18	10.5	11.4	11.9	95.07	95.10	95.35	95.00

**Table 4:** Optimized parameters of the proposed MIMO antenna.

Parameter	L_m1_	L_m2_	W_m1_	W_m2_	S_m1_
Value (mm)	23.23	8.91	5.86	2.26	1.69

**Table 5:** Computed ECC and DG of the proposed 2×1 MIMO antenna.

Frequency (GHz)	ECC		DG (dB)	
	Simulated	Measured	Simulated	Measured
2.45	<0.001	<0.001	>9.99	>9.99
3.60	<0.001	<0.001	>9.99	>9.99
5.80	<0.001	<0.001	>9.99	>9.99

**Table 6:** Comparisons of some multiband quasi-Yagi antennas.

Ref.	Center Frequency (GHz)	Gain (dBi)	Antenna Size ($\lambda_{0}\times \lambda_{0}$)	Front–Back Ratio (dB)
[9]	1.8, 2.4, 3.5	NG	0.31 × 0.37	~10
[10]	1.9, 2.4, 3.6	<4.8	0.32 × 0.32	>10
[11]	13.5, 30, 60	<7	0.23 × 0.23	>4.5
[16]	3.97, 5.52, 8.22	<5	0.38 × 0.24	>9
[17]	2.02, 5.37, 9.64	~5, ~7, NG	NG	NG
[21]	1.9, 2.5, 3.5	6.29, 4.63, 6.77	0.44 × 0.51	>10.8
This work (Single MBLD)	2.45, 3.60, 5.8	6.70, 7.4, 7.5	0.65 × 0.49	>13.0
This work (Five MBLDs)	2.45, 3.60, 5.8	10.1, 11.8, 11.9	1.63 × 0.49	>18.0

## Data Availability

All data that support the findings of the study has been included into the manuscript.

## Abbreviations

The following abbreviations are used in this manuscript:

MBLD	Multi-Branch Line Director
MIMO	Multiple-Input Multiple-Output
RF	Radio Frequency
VSWR	Voltage Standing Wave Ratio
FBR	Front-to-Back Ratio
IoT	Internet of Things
PCB	Printed Circuit Board
EM	Electromagnetics
